# Development of a Toxic Lead Ionic Sensor Using Carboxyl-Functionalized MWCNTs in Real Water Sample Analyses

**DOI:** 10.3390/s22228976

**Published:** 2022-11-20

**Authors:** Hadi M. Marwani, Jahir Ahmed, Mohammed M. Rahman

**Affiliations:** 1 Chemistry Department, Faculty of Science, King Abdulaziz University, P.O. Box 80203, Jeddah 21589, Saudi Arabia; 2 Center of Excellence for Advanced Materials Research (CEAMR), King Abdulaziz University, P.O. Box 80203, Jeddah 21589, Saudi Arabia; 3 Promising Centre for Sensors and Electronic Devices (PCSED), Advanced Materials and Nano-Research Centre, Najran University, P.O. Box 1988, Najran 11001, Saudi Arabia; 4 Department of Chemistry, Faculty of Science and Arts, Najran University, P.O. Box 1988, Najran 11001, Saudi Arabia

**Keywords:** f-MWCNT, lead ions, ICP-OES, selectivity, real samples, environmental safety

## Abstract

Functional multiwall carbon nanotubes (f-MWCNTs) are of significant interest due to their dispersion ability in the aqueous phase and potential application in environmental, nanotechnology, and biological fields. Herein, we functionalized MWCNTs by a simple acid treatment under ultra-sonification, which represented a terminal or side-functional improvement for the fabrication of a toxic lead ion sensor. The f-MWCNTs were characterized in detail by XRD, Raman, XPS, BET, UV/vis, FTIR, and FESEM-coupled XEDS techniques. The analytical performance of the f-MWCNTs was studied for the selective detection of toxic lead ions by inductively coupled plasma-optical emission spectrometry (ICP-OES). The selectivity of the f-MWCNTs was evaluated using several metal ions such as Cd^2+^, Co^2+^, Cr^3+^, Cu^2+^, Fe^3+^, Ni^2+^, Pb^2+^, and Zn^2+^ ions. Lastly, the newly designed ionic sensor was successfully employed to selectively detect lead ions in several environmental water samples with reasonable results. This approach introduced a new technique for the selective detection of heavy metal ions using functional carbon nanotubes with ICP-OES for the safety of environmental and healthcare fields on a broad scale.

## 1. Introduction

Recently, carbon nanotubes (CNTs) have become an interesting material in fundamental research due to their versatile applications such as miniaturized bio-electronic devices and the detection of carcinogenic metallic ions. Their small dimensions, as well as the extraordinary physical properties of functionalized multiwalled carbon nanotubes (f-MWCNTs), has made them an excellent material for multipurpose applications [1]. Since the discovery of CNTs in 1991, they have been introduced as a new subject in many studies in chemical, physical, sensor, adsorption, and material science fields owing to their unique structural, mechanical, electronic, and electrochemical properties [2,3,4,5,6]. Based on their diameter and chirality, CNTs can electrically behave either as a metal or a semiconductor [7]. Functionalized multiwalled carbon nanotubes (f-MWCNTs) have drawn huge attention in the last few years. Research works regarding the promising application of f-MWCNTs are considered to be crucial. Recent developments in sensitive and effective techniques have become important to monitor toxic metals in the environment. Several analytical techniques have been utilized to determine heavy metals from aqueous systems such as atomic absorption spectrometry [8], inductively coupled plasma-optical emission spectrometry (ICP-OES) [9], anodic stripping voltammetry [10], ion chromatography [11], and electrochemical methods [12,13]. However, metal ions cannot be directly determined from aqueous systems by these analytical methods, especially with ultra-low levels of metal ions, because of poor sensitivity and selectivity. Consequently, an appropriate separation step is often necessary before the detection of metal ions [14]. Many extraction techniques of the analyte of interest can be implemented before the detection of metal ions with suitable analytical techniques such as liquid–liquid extraction [15], ion exchange [16], co-precipitation [17], cloud point extraction [18], and solid-phase extraction (SPE) [19,20]. Among them, SPE is recognized as the most popular method due to less solvent usage, the disposal costs, and the extraction time in the sample preparation. Due to the attractiveness of SPE in selective analyte extractions, many adsorbents have been used, including alumina [21], C18 [22], molecular imprinted polymers [23], cellulose [24], silica gel [25], activated carbon [26,27], and CNTs [28].

Nanoscale materials have also attracted considerable interest owing to their prospective application in fabricating opto-electronics, electro-analytics, the selective detection of metal ions, opto-electronics, biological devices, nanocomposites, electron-field emission sources for emission exhibits, chemical detections, and surface-enhanced Raman properties [29,30,31]. They have typical morphological structures that are composed of a number of regular phases, with geometrically controlled metals as well as oxide atoms along the axes. Here, we prepared functionalized multiwalled carbon nanotubes; in the preparation method, there were several advantages such as a low-temperature preparation and an accurate control of the stoichiometry. They were also easy to handle with a one-step reaction and a high porosity as well as a high surface area. The optical, morphological, electrical, and chemical properties of nanoscale carbon nanomaterials are of huge significance from scientific aspects [32,33,34]. In nanostructure materials, the doped material has a large band gap (E_bg_), which, in its non-stoichiometric form, exhibits a non-insulating nature. Non-stoichiometry, mostly oxygen vacancies, forms its conducting nature. The formation energy of oxygen vacancies and metal interstitials in metal oxides is very low; thus, these defects eagerly form, resulting in the experimental elevated conductivity of the nanoscale substrates [35,36]. Electrical properties such as the charge carrier concentration and conductivity can further be enhanced by extrinsic transition metallic dopants into the nanomaterials. There are many reports in the literature that highlight the sensing properties of pure and doped/undoped materials [37,38]. However, to date, there are no reports focusing on the adsorption properties of the uptake of metal ions (lead toxic ions) on carboxylic functional carbon nanotubes in the aqueous phase.

Generally, carbon nanoscale materials such as carbon nanotubes (CNTs), graphene (G), fullerene, and carbon nanofibers play a significant role in the detection of analytes for the safety of environmental and healthcare fields. Amongst them, multiwalled carbon nanotubes (MWCNTs) are a promising material for the fabrication of electrochemical sensors in room conditions owing to their large specific surface area, excellent electrical conductivity, rapid electrode kinetics, and high chemical stability [39]. To improve the sensor probe performance (such as the selectivity, long-term stability, linear dynamic range, and sensitivity), composites of two or more materials and the functionalization of carbon nanotubes have been explored for electrode modifications to study the potential synergistic effects. A higher peak current could be obtained through the proper selection of composite components with functionalized carbon materials, compared with each material or unfunctionalized carbon material working alone [40]. Until today, several methods (e.g., hydrothermal, solution, sol–gel, and sono-chemical) have been utilized for the preparation of functional carbon composites. 

Therefore, in this work, we devoted ourselves to investigating the analytical ability of f-MWCNTs as the selective extractor of lead ions before detection with ICP-OES. The selectivity of the f-MWCNTs using several metal ions such as Cd^2+^, Co^2+^, Cr^3+^, Cu^2+^, Fe^3+^, Ni^2+^, Pb^2+^, and Zn^2+^ was investigated to study the efficiency of the f-MWCNTs in the selective detection of metal ions. The selectivity study data revealed that the f-MWCNTs showed the most selective behavior toward lead ions than other metal ions. The adsorption capacity for Pb^2+^ ions was experimentally found to be ~77.12 mgg^−1^. We also investigated the analytical parameters. The adsorption isotherm results also established that this adsorption process formed a monolayer onto a perfectly homogeneous surface with a finite number of identical sites. For the first time, a new route for the uptake of selective heavy metal ions with functionalized carbon materials with ICP-OES for the safety of environmental and healthcare fields on a broad scale is introduced.

## 2. Experiment

### 2.1. Materials and Methods

For the experiment, we purchased reagents (reagent grade) of 97% H_2_SO_4_, 60% HNO_3_, 98% Triton X-100, and 30% H_2_O_2_ from the Showa Chemical Company (Tokyo, Japan); these were used as received without further purification. We used deionized (DI) distilled water (resistivity > 18.0 MΩcm) to prepare he different solutions. We purchased standard stock solutions of 1000.0 mgL^−1^ Cd^2+^, Co^2+^, Cr^3+^, Cu^2+^, Fe^3+^, Ni^2+^, Pb^2+^, and Zn^2+^ from Sigma-Aldrich (USA). All reagents used in this work were highly pure. We recorded the FTIR spectrum of the f-MWCNTs using a Bruker spectrophotometer. The morphology of the f-MWCNTs was studied by an FESEM instrument (JSM-7600F, Tokyo, Japan). An energy dispersive X-ray analysis (XEDS) was examined for the f-MWCNTs using an FESEM-coupled XEDS from JEOL (Tokyo, Japan). The X-ray photoelectron spectroscopy (XPS) measurements were executed on a Thermo Scientific K-Alpha KA1066 spectrometer (NY, USA) for the f-MWCNTs. In the optical analysis, a monochromatic AlKα X-ray radiation source was used as the excitation source; the beam spot size was kept at 300.0 μm. The spectra were recorded in the fixed analyzer transmission mode, where the pass energy was kept at 200.0 eV. The scanning of the spectra was performed at pressures less than 10−8 Torr. The XRD spectrum was recorded using an X’Pert Explorer PANalytical diffractometer equipped with Cu-Kα1 radiation (λ = 1.5406 nm) by a generator voltage (~40.0 kV) and current (~35.0 mA). The UV/vis spectrum of the f-MWCNTs was recorded using Lamda-950 (Perkin Elmer, Berlin, Germany). The ICP-OES investigations were performed using a Perkin Elmer ICP-OES model Optima 4100 DV (NY, USA). The BET analysis for the pore-size distributions and the surface area of the f-MWCNTs for different nitrogen gas loadings was performed with a Quantachrome. The parameters for the BET analysis are given here.

Sample weight: 0.0075 g; outgas time, 4.0 h; analysis gas, N_2_; pressure tolerance, 0.100/0.100 (ads/des); analysis time, 602.4 min; sample volume, 0.0025 cc; outgas temp, 250.0 °C; bath temp, 77.3 K; equil. time, 60/60 (ads/des); sample density, 3.0 g/cc, equil. timeout, 240/240 s (ads/des). DFT: N_2_ at 77.0 K on carbon (slit pore, NLDFT equilibrium model); release pressure range, 0.0000–1.0000. Adsorbate: nitrogen gas; cross section, 16.200; liquid density, 0.806 g/cc.

We optimized the ICP-OES instrument every day immediately before the measurements. It was operated as per the manufacturer’s recommendations. The ICP-OES spectrometer was used with the following parameters: FR power, 1300 kW; frequency, 27.12 MHz; demountable quartz torch, Ar/Ar/Ar; plasma gas (Ar) flow, 15.0 Lmin^−1^; auxiliary gas (Ar) flow, 0.2 Lmin^−1^; nebulizer gas (Ar) flow, 0.8 Lmin^−1^; nebulizer pressure, 2.4 bar; glass spray chamber according to Scott (Ryton) sample pump flow rate, 1.5 mLmin^−1^; integration time, 3 s; replicates, 3; wavelength range of monochromator, 165–460 nm. Selected metal ions were measured at wavelengths of 228.80 nm for Cd(II), 238.90 nm for Co(II), 267.72 nm for Cr(III), 327.39 nm for Cu(II), 259.94 nm for Fe(III), 221.65 nm for Ni(II), 220.35 nm for Pb(II), and 206.20 nm for Zn(II). 

### 2.2. Preparation and Purification of the f-MWCNTs

Generally, carbon nanotubes are pure although a few nanoparticles might be present in the as-procured sample as a by-product. MWCNTs can exist in isolated units or as nanotubes in a bundled arrangement; we did not take any steps to separate them. The refinement of common CNTs is important as most of the CNT applications require a high-purity material. An acid treatment is the most usual means to purify CNTs and has been recognized as the first step in the numerous purification schemes. A nitric acid treatment is commonly used for removing the metal catalysts and amorphous carbons [41]. Additionally, it also oxidizes the terminal carbon atoms. Sonicating the CNTs in nitric acid opens the terminal carbons [42]; hence, -COOH groups at the ends or the defect sites of CNTs are introduced [43]. We functionalized the MWCNTs with -COOH groups using a nitric acid treatment. Briefly, we ultra-sonically dispersed the MWCNTs in 5.0 M nitric acid for 6.0 min. We then diluted it using a large volume of water and added a little Triton X-100 to enhance the solubility. We continued the sonication process until it became a black solution. Later, we collected the f-MWCNTs by filtration using a 0.2 µm diameter film. We repeated these steps twice. The evidence for the formation of a functional carboxyl group on the f-MWCNTs was exhibited by FTIR spectroscopy (a broad peak appeared at 1712 cm^−1^). 

### 2.3. Sample Preparation and Procedure

Stock solutions of Cd^2+^, Co^2+^, Cr^3+^, Cu^2+^, Fe^3+^, Ni^2+^, Pb^2+^, and Zn^2+^ were prepared using 18.2 MΩ·cm DI water and stored at 4.0 °C in a dark place. In the selectivity study of the f-MWCNT system toward different metal ions, we prepared 5.0 mgL^−1^ of a standard solution for each metal ion and adjusted the pH to 5.0 using an acetate buffer. We mixed 25.0 mg of the f-MWCNTs with each of the standard solutions separately. We used a pH value of 5.0 for all metal ions to avoid precipitation, particularly for Fe^3+^ due to the formation of an Fe(OH)_3_ precipitate at higher pH values. To investigate the Pb^2+^ adsorption capacity, standard solutions of 0, 5.0, 10.0, 15.0, 20.0, 25.0, 30.0, 50.0, 70.0, 85.0, 125.0, and 150.0 mgL^−1^ were also prepared as mentioned above; the pH value was adjusted to 5.0 and separately mixed with 25.0 mg f-MWCNTs. We shook all mixtures for 1.0 h at 150 rpm in ambient conditions. The effect of the contact time on the uptake of the f-MWCNTs for the Pb^2+^ ion was investigated using the same batch conditions but at different equilibrium periods (2.5, 5, 10, 20, 30, 40, 50, and 60 min).

## 3. Results and Discussion

### 3.1. Evaluation of the Spectral Analysis

**Figure 1:**
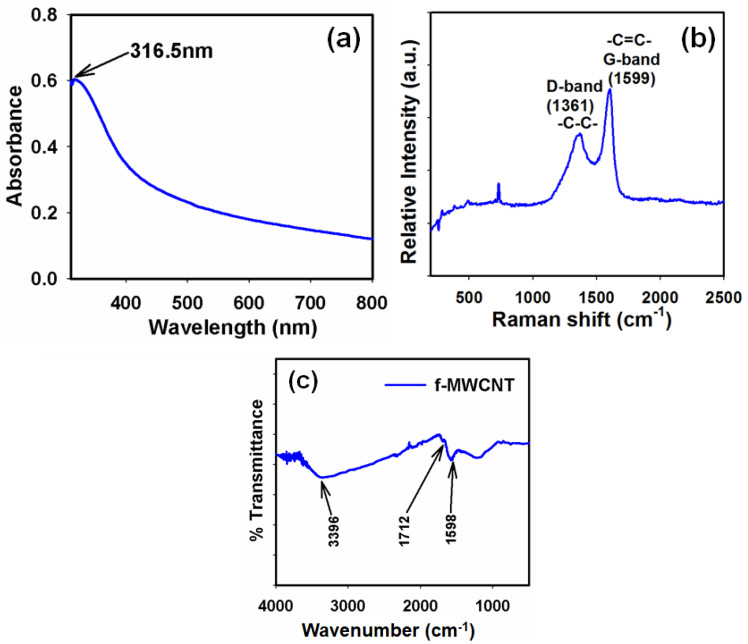
Typical (**a**) UV/vis, (**b**) Raman, and (**c**) FTIR spectra of the f-MWCNTs.

To evaluate the physical activity of the f-MWCNTs, we recorded the optical absorption spectra in the range of 200.0–800.0 nm in ambient conditions. In the UV/vis spectroscopic technique, the outer electrons from the active materials undergo an electronic transition from low to high energy states by absorbing the radiant energy [44,45]. Herein, we used the optical absorption band to estimate the band gap energy (*E*_bg_) of the f-MWCNTs. It was revealed that the maximum absorption of the radiation occurred at ~316.5 nm (Figure 1a). The *E*_bg_ was calculated as ~3.9 eV based on the absorption band of the f-MWCNTs using Equation (1):

(1) $$E_{\mathrm{bg}}=\frac{1240}{\lambda }\left(\mathrm{eV}\right)$$

where λ is the wavelength (~316.5 nm) at which the maximum absorption occurred. The absence of additional peak(s) related to impurities indicated that the functionalization process controlled the crystallinity of the f-MWCNTs [46].

The Raman spectroscopic technique is a versatile tool to characterize CNTs. All allotropes of carbon (such as fullerenes, CNTs, amorphous carbon, and polycrystalline carbon) are Raman-active [47]. The position, width, and relative intensity of the bands are modified based on the types of carbon [48,49,50,51]. The tangential mode (TM) in the range of 1400–1700 cm^−1^ provides information regarding the electronic properties. The D-band that appears at 1361 cm^−1^ provides information regarding the disordered carbon. The D-band to TM band ratio is a qualitative measure for the formation of undesirable forms of carbon [52,53]. Herein, we used a 788 nm (semiconductor sapphire laser) excitation. This provided the most direct evidence of the f-MWCNTs directly observed from the Raman spectrum. In the Raman spectrum of the f-MWCNTs (and as shown in Figure 1b), the G-band at 1599 cm^−1^ appeared from the graphitic sheet structures [54,55] and the D-band at 1361 cm^−1^ correlated with the defects in MWCNTs [54,55]. Therefore, we concluded that there was no change in the physical structure of the MWCNTs during the functionalization process except for the opened ends.

We also recorded the FTIR spectrum of the f-MWCNTs to identify the different functional groups attached to the f-MWCNT surface [56,57,58]. FTIR spectroscopy has been extensively used in the structural determination of molecules. Figure 1c shows comparative FTIR data for the refluxed samples. As observed in the prepared sample, there was a signal with a small C-C stretch (1598 cm^−1^). With the acid treatment, a number of new peaks appeared. The bands due to the C=O stretch were prominently seen at 1712 cm^−1^ for the carboxylated MWCNTs. The sample refluxed in 3:1 H_2_SO_4_:HNO_3_ acid for 6–7 h showed a distinct band at 1712 cm^−1^, which could be assigned to the acid carbonyl-stretching mode (Figure 1c). Another band exhibited in this functionalized sample was at 3396 cm^−1^, which was characteristic of O-H stretches. C-C vibrations occurred due to internal defects and the O-H vibration was associated with the amorphous carbon because amorphous carbon easily forms a bond with atmospheric air. However, the intensity of this O-H peak was relatively lower and showed that a lesser amount of amorphous carbon formed during the growth [59]. The peak at 1598 cm^−1^ could be associated with the stretching of the carbon nanotube backbone [60]. Thus, the evidence for the formation of a functional carboxyl group (with a peak at 1712 cm^−1^) on the MWCNTs was investigated by FTIR spectroscopy. 

### 3.2. Evaluation of the Structural and Morphological Analysis

**Figure 2:**
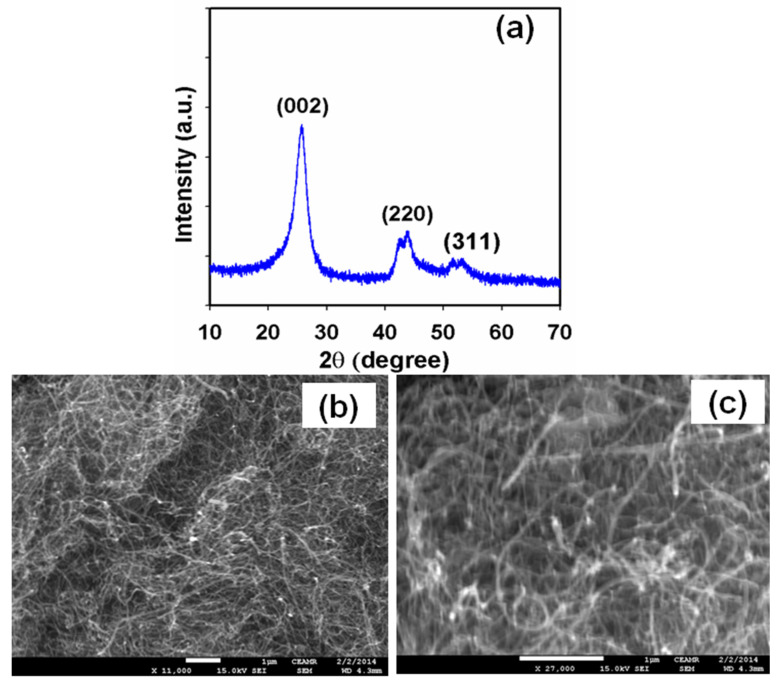
Typical (**a**) powder XRD and (**b**,**c**) low to high magnified FESEM images of the f-MWCNTs.

As in recent articles [61,62], the diffraction patterns of the wall of the f-MWCNTs also displayed a peak of 26.1° at 2θ as shown in Figure 2a. This peak could be related to the (002) lattice plane [48,52]. The XRD pattern of the f-MWCNTs was similar to that of the pristine MWCNTs [53]. Therefore, we concluded that the f-MWCNTs still possessed the same cylindrical-walled structure and inter-planner spacing as the pristine MWCNTs even after the functionalization process. Hence, the structural property of the f-MWCNTs remained the same even after the acid treatment, as confirmed by the previous XRD investigation [63]. The diameter of the f-MWCNTs was also calculated using the Scherrer Equation [64,65,66,67]:

D = 0.9λ/(βcos θ)(2)

where λ is the wavelength of X-ray radiation and β is the full width at half maximum (FWHM) of the peaks at the diffracting angle θ. The average diameter of the f-MWCNTs was obtained as ~11.2 nm. 

High-resolution FESEM images of the f-MWCNTs (low and higher magnification) are presented in Figure 2b,c. The FESEM images revealed that aggregated functional materials existed in tube shapes. The average diameter of the f-MWCNTs was estimated to be 5.0 to 20.0 nm, which was close to ~11.9 nm. The FESEM images showed that the f-MWCNTs had a regular tube shape with high-density material. Therefore, the nitric acid treatment of the MWCNTs caused severe etching on the graphitic surface, producing shorter tubes with a huge number of disordered sites [68]. The analysis of the FESEM images of the f-MWCNTs allowed a trustworthy length measurement of the nanotubes; these measured values were between 5.0 and 20.0 nm, identical to those for pristine MWCNTs according to the literature [69].

### 3.3. Evaluation of the Elemental Analysis

**Figure 3:**
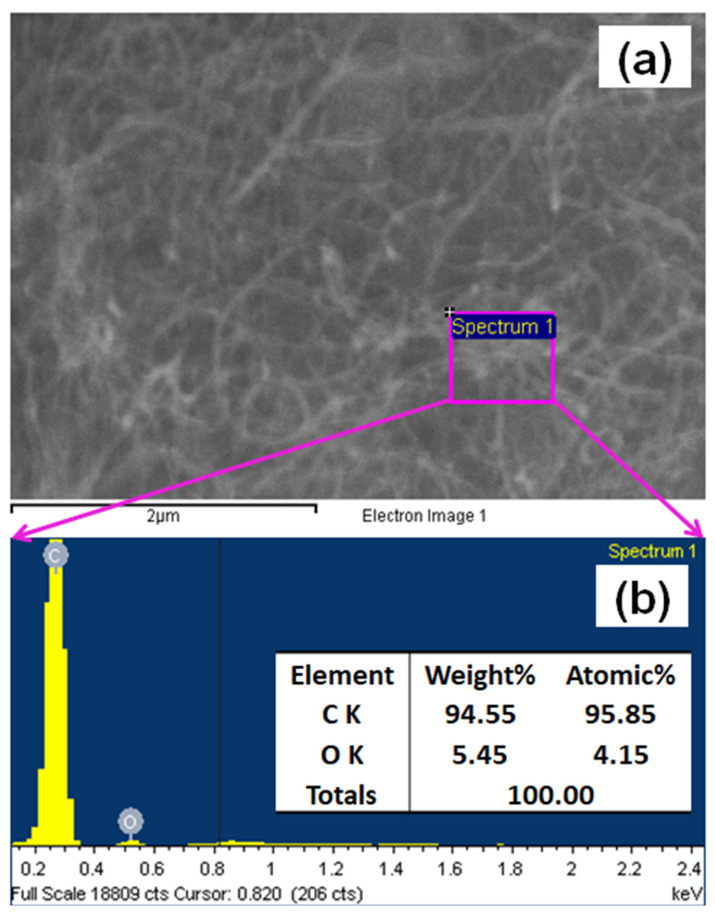
Typical XEDS measurements for the f-MWCNTs: (**a**) selected area for the XEDS measurements; (**b**) XEDS spectrum with elemental compositions.

The XEDS investigation confirmed the existence of C and O elements in the f-MWCNTs, as shown in Figure 3a. The weight percent compositions of C and O were 94.55% and 5.45%, respectively. The XEDS spectrum and corresponding elemental analysis data are presented in Figure 3b. The absence of any other extra peaks in the XEDS spectrum confirmed the purity of the f-MWCNTs. After the functionalization of the MWCNTs, the as-obtained final product—f-MWCNTs—contained an oxygen element in the nanotubes, which confirmed the formation of carboxylic groups (-COOH) in the carbon nanotubes.

### 3.4. Evaluation of the Binding Energy Analysis

**Figure 4:**
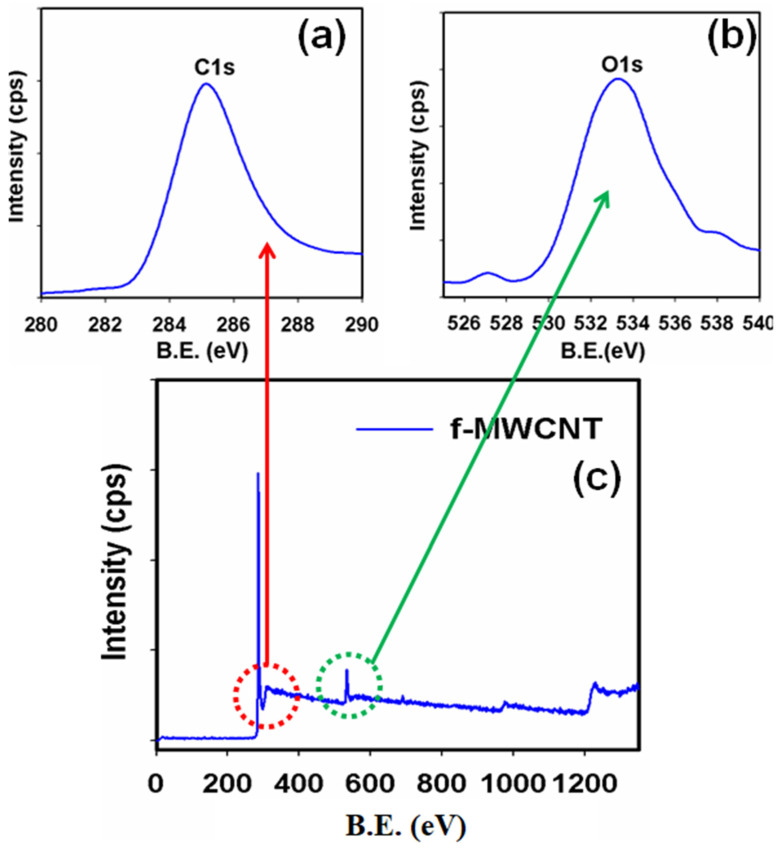
Typical XPS spectra showing the binding energy (B.E.) for (**a**) C1s level, (**b**) O1s level, and (**c**) f-MWCNT level acquired from MgKα1 radiation.

XPS investigations were performed to further confirm the elemental compositions and chemical states of the different elements present in the f-MWCNTs. Figure 4a shows the spin–orbit peak of the C1s binding energy that appeared at around 285.1 eV, which was well-matched with the literature [48,52,53,70]. The asymmetric XPS peak for O1s appeared at 532.7 eV, as shown in Figure 4b, and confirmed the existence of oxygen in the f-MWCNTs [71,72,73,74]. The full-scan XPS spectrum of the f-MWCNTs, as shown in Figure 4c, clearly showed two distinct peaks for C1s and O1s. Therefore, we concluded that the f-MWCNTs contained only two different elements; this was also supported by the XRD, Raman, and FTIR investigations.

### 3.5. Evaluation of the BET Analysis

**Figure 5:**
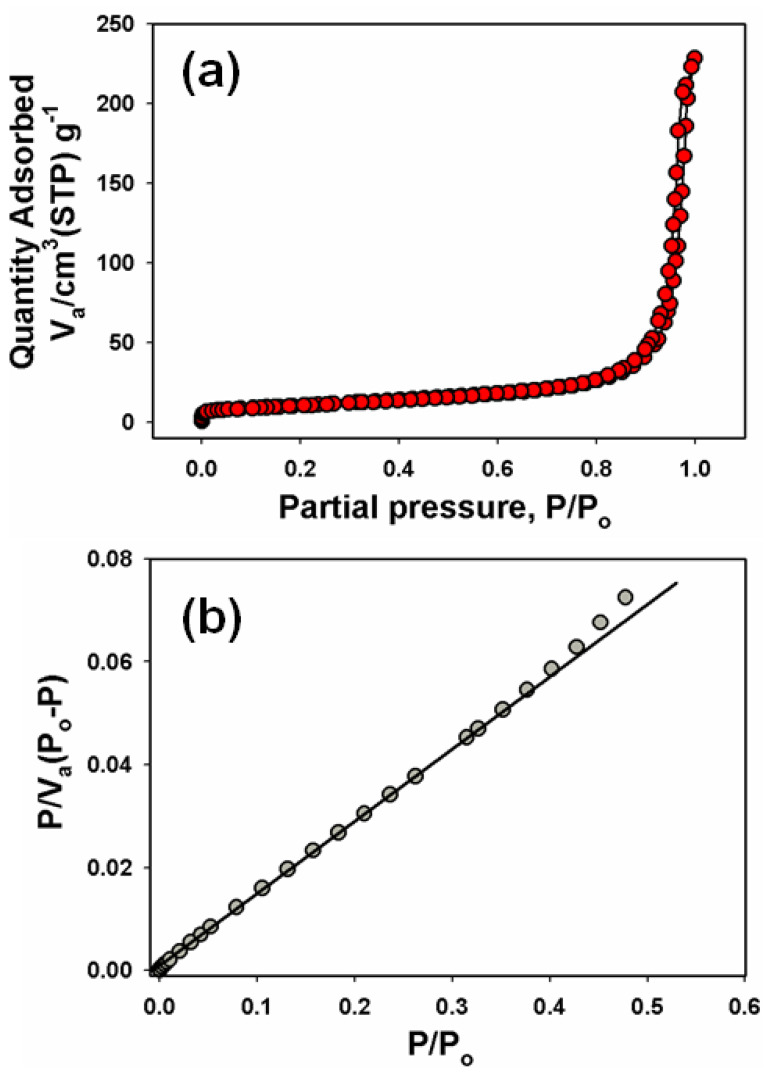
BET analysis: (**a**) surface area and (**b**) pore−size distribution of the f-MWCNTs for different nitrogen gas loadings.

The Brunauer–Emmett–Teller (BET) theory explains the physical adsorption of nitrogen gas on mesoporous functionalized carbon nanotubes and thus measures the specific surface area of f-MWCNTs. The average pore diameter and specific surface area (BET: surface area and pore volume) were measured for the f-MWCNTs using a Quantachrome NOVA 1000 (NY, USA). To observe whether any changes to the physical structure of the f-MWCNTs occurred, we investigated the specific surface area (adsorption/desorption isotherms) and the pore-size distribution using a multipoint BET analysis. Figure 5a shows that the specific surface area of the f-MWCNTs was 222.450 m^2^/g and Figure 5b shows the pore-size distribution. This high surface area originated from the formation of nanopores accessible to nitrogen gas, leading to an amplified capacitance of the f-MWCNTs. The experimental results showed that changes in the conditions during the acid treatment of MWCNTs greatly affected the specific surface area and pore-size distribution of the f-MWCNTs, as displayed in Figure 5 (adsorption/desorption isotherms in Figure 5a and size distribution plot in Figure 5b).

### 3.6. Lead Ion Detection Using f-MWCNTs with ICP-OES (Static Adsorption Method)

#### 3.6.1. Selectivity Study of the f-MWCNTs

**Figure 6:**
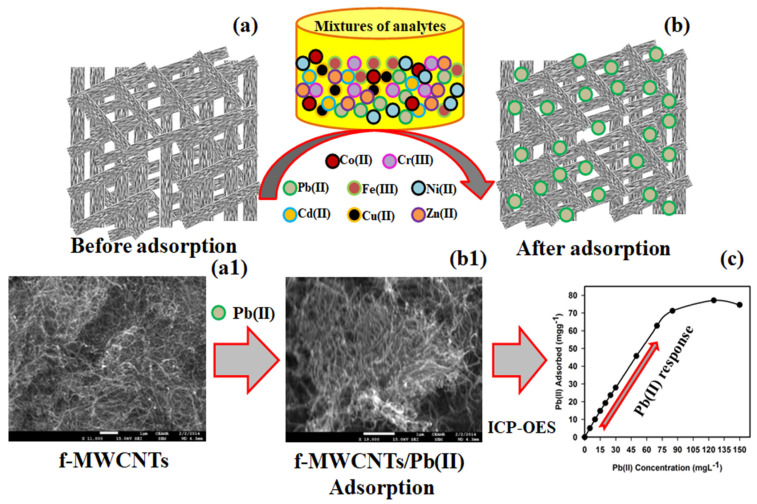
Schematic representation and FESEM images of (**a**) before and (**b**) after Pb^2+^ adsorption on the f-MWCNTs. (**c**) Pb^2+^ ion detection with the ICP-OES method using the functionalized MWCNTs.

The selectivity investigations for the f-MWCNT phase toward different metal ions were performed using the batch adsorption method. The selectivity was also investigated using the distribution coefficient (*K_d_*) for the f-MWCNT phase, which could be calculated by Equation (3) [75]:

*K*_d_ = (*C*_o_ − *C*_e_/*C*_e_) × (*V*/*m*) (3)

where *C*_o_ and *C*_e_ are the initial and final concentrations before and after filtration with the f-MWCNTs, respectively, *V* is the volume (mL), and *m* is the weight of the f-MWCNT phase (g). Table 1 shows the calculated *K_d_* values for all metal ions, which revealed that the Pb^2+^ ion had a greater *K_d_* value than the other metal ions. A scheme representing the Pb^2+^ ion adsorption onto the surface of the f-MWCNTs with comparative schemes using real FESEM images is displayed in Figure 6, which shows the FESEM images of the f-MWCNTs before (Figure 6(a,a1)) and after (Figure 6(b,b1)) the adsorption of the Pb^2+^ ions. The FESEM image of the f-MWCNTs again revealed an aggregated tube-shaped morphology with a high density. A dwindle image was observed after the Pb^2+^ adsorption onto the f-MWCNT surface and the edges of the tubes were not clearly visible (Figure 6(b1)). We also proposed that approximately all the Pb^2+^ ions adsorbed onto the aggregated functional nanotubes were composed of a large surface area. The lead ions were adsorbed onto the functional as well as the defect sites of the f-MWCNTs. With the ICP-OES method, the detection responses of the Pb^2+^ ions using the f-MWCNTs were clearly demonstrated; these are presented in Figure 6c.

Therefore, based on the above results, we concluded that the selectivity of the f-MWCNT phase toward the Pb^2+^ ions was greater than all other metal ions. Thus, the f-MWCNT phase could selectively detect Pb^2+^ ions, providing that the mechanism of adsorption was an electrostatic attraction or a complex formation. 

#### 3.6.2. Static Adsorption Capacity of the f-MWCNTs

**Figure 7:**
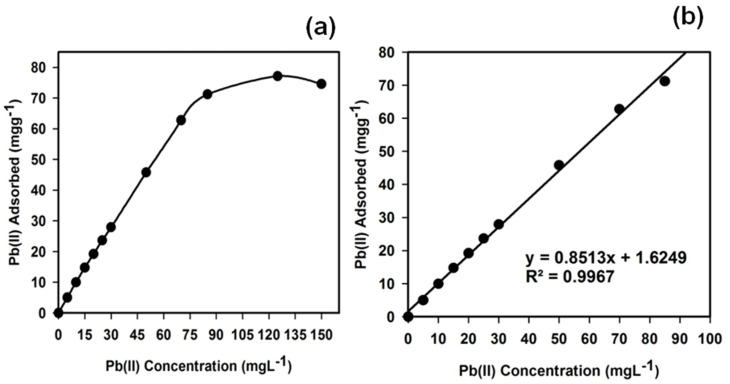
(**a**) Adsorption profile and (**b**) calibration curve (for sensitivity) of Pb^2+^ ions in 25.0 mg of the f−MWCNT phase in relation to the concentration at pH 5.0 and 25.0 °C.

To estimate the uptake capacity of the Pb^2+^ ions onto the f-MWCNT phase, 25.0 mL Pb^2+^ ion samples with varying concentrations (0–150.0 mgL^−1^) were adjusted to pH 5.0 and separately mixed with 25.0 mg of the f-MWCNTs. These mixtures were mechanically shaken for 1.0 h in ambient conditions. The static adsorption capacity was calculated using Equation (4):

(4) $$q_{e}=\frac{(C_{o}-C_{e})V}{m}$$

where *q*_e_ is the adsorbed Pb^2+^ ions onto the f-MWCNT phase (mgg^−1^); *C*_o_ and *C*_e_ represent the initial and equilibrium concentrations of the Pb^2+^ ions (mgL^−1^), respectively; *V* refers to the volume (L); and *m* is the weight of the f-MWCNT phase (g). Figure 7 displays the Pb^2+^ ion adsorption profile and the calibration curve based on the experimental adsorption isotherms. The adsorption capacity and sensitivity of the f-MWCNTs in the Pb^2+^ ion detection reached 77.12 mgg^−1^ and 0.8513 Lg^−1^, respectively, which were comparable with the literature regarding Pb^2+^ ion detection by other methods (32.75 [76], 49.9 [77], 54.48 [78], 82.66 [79], 90.25 [80], 97.08 [81], and 114.05 mgg^−1^ [82]) (Table 2). The linear dynamic range from the calibration plot was obtained as 0 to 85.0 mgL^−1^. 

#### 3.6.3. Adsorption Isotherm Models of the f-MWCNTs

The experimental results for the adsorption of Pb^2+^ ions onto the f-MWCNT phase were studied by common models to interpret the equilibrium isotherm data. The Langmuir equation is effective in monolayer sorption onto a completely homogeneous surface with a finite number of identical sites and a negligible interaction between the adsorbed molecules. The Langmuir adsorption isotherm model is presented by Equation (5) [83]:

*C*_e_*/q*_e_ = (*C*_e_*/Q*_o_) + 1/*Q*_o_*b* (5)

where *C*_e_ is the equilibrium concentrations of the Pb^2+^ ions (mgmL^−1^) and *q*_e_ is the adsorbed Pb^2+^ ions by the f-MWCNTs (mgg^−1^). The symbols *Q*_o_ and *b* refer to the Langmuir constants related to the adsorption capacity (mgg^−1^) and energy of adsorption (Lmg^−1^), respectively. These constants can be calculated using the linear plot of *C*_e_/*q*_e_ vs. *C*_e_, where the slope and intercept are equal to 1/*Q*_o_ and 1/*Q*_o_*b*, respectively. The important features of the Langmuir adsorption isotherm model can be expressed using an equilibrium parameter (*R_L_*), where *R_L_* = 1/(1 + *bC*_o_). Here, *b* is the Langmuir constant that specifies the nature of adsorption and the shape of the isotherm; *C*_o_ refers to the initial concentration of the analytes. The *R_L_* value offers information regarding the type of adsorption isotherm; its value lies between 0 and 1, suggesting an encouraging adsorption [84].

**Figure 8:**
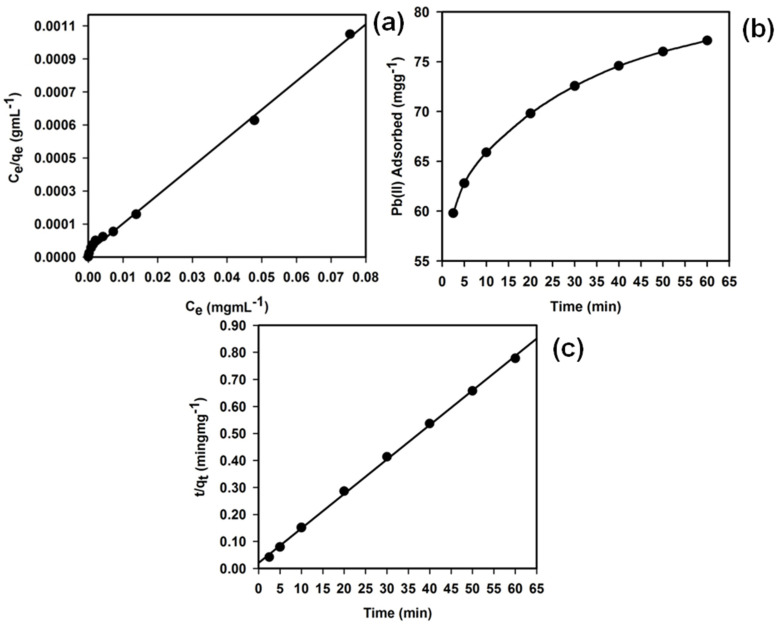
(**a**) Langmuir adsorption isotherm model; (**b**) effect of the shaking time on adsorption; (**c**) pseudo−second−order adsorption kinetic model of Pb^2+^ ion adsorption upon the 25.0 mg f-MWCNT phase at pH 5.0 and 25.0 °C. Adsorption experiments were performed using varying concentrations (0–150.0 mgL^−1^) of Pb^2+^ ions under static conditions.

The experimental isotherm data were the best fit with the Langmuir equation (Figure 8a) based on the least squares fit, confirming the validity of the Langmuir adsorption isotherm model for this adsorption process. This revealed that the adsorption process in this study was primarily a monolayer formation onto a homogeneous adsorbent surface. Langmuir constants *Q*_o_ and *b* were obtained as 77.30 mgg^−1^ and 0.57 Lmg^−1^, respectively. The regression coefficient for the Langmuir model was obtained as *R*^2^ = 0.9970 during the Pb^2+^ ion adsorption onto the f-MWCNTs. Furthermore, the calculated Pb^2+^ ion adsorption capacity (77.30 mgg^−1^) obtained from the Langmuir equation was consistent with that of the experimental isotherm study (77.12 mgg^−1^). The *R_L_* value of the Pb^2+^ ion adsorption onto the f-MWCNTs was 0.01, suggesting a very favorable adsorption process based on the Langmuir classical adsorption isotherm model.

#### 3.6.4. Effect of the Shaking Time

The effect of the contact time was examined to confirm the applicability of the f-MWCNTs to the selective adsorption of Pb^2+^ ions and to estimate the time required to establish the equilibrium. A batch process was employed for varying contact times (2.5 to 60.0 min) using a fixed concentration of 125.0 mgL^−1^ Pb^2+^ ions, as illustrated in Figure 8b. A close investigation, as presented in Figure 8b, demonstrated that the adsorption of Pb^2+^ ions onto the f-MWCNT phase was significantly improved with the increasing contact time. Over 65.0 mgg^−1^ Pb^2+^ ions were adsorbed onto the f-MWCNTs in just 10 min of the equilibrium period. The loading capacity of the Pb^2+^ ions was also raised to more than 72.0 mgg^−1^ after 30 min until the maximum adsorption of the Pb^2+^ ions onto the f-MWCNTs was reached after 60 min. Therefore, we concluded that the equilibrium kinetics for the adsorption of Pb^2+^ ions onto the f-MWCNT phase were very fast.

#### 3.6.5. Kinetic Model Analysis

To obtain the kinetic adsorption parameters, we investigated several kinetic models. Kinetic models were used to check how properly they fitted with the experimental data, where the regression coefficient (*R^2^*) was a measure of agreement between the experimental data and the models. The kinetics for pseudo-second-order adsorption were presented by Equation (6):

*t*/*q*_t_ = 1/*υ*_o_ + (1/*q*_e_)*t* (6)

where *υ*_o_ = *k*_2_$q_{e}^{2}$ denotes the initial adsorption rate (mgg^−1^ min^−1^); *k*_2_ (gmg^−1^ min^−1^) represents the rate constant of the adsorption; *q*_e_ (mgg^−1^) is the amount of metal ions adsorbed at the equilibrium; and *q*_t_ (mgg^−1^) refers to the amount of metal ions on the adsorbent surface at any time *t* (min). The parameters *q*_e_ and *υ*_o_ could be obtained from the slope and intercept of a plot of *t*/*q*_t_ vs. *t*, respectively [85].

The adsorption kinetics data were well-matched with the pseudo-second-order model, suggesting that the kinetics of Pb^2+^ ion adsorption onto the f-MWCNTs obeyed pseudo-second-order kinetics (Figure 8c). The *R*^2^ value (0.998) also supported that the pseudo-second-order model was better than other kinetic models. Parameters *υ*_o_, *q*_e_, and *k_2_* were calculated as 47.84 mgg^−1^ min^−1^, 78.29 mgg^−1^, and 0.01 gmg^−1^ min^−1^, respectively. The value of *q*_e_ was consistent with the results of the adsorption isotherms, supporting the validity of the Langmuir adsorption isotherm model. In this approach, we found the highest results to the lead ion uptake were with the ICP-OES technique with carboxylic functional MWCNTs compared with other nanostructure materials [86,87,88,89,90,91,92,93,94,95,96].

#### 3.6.6. Real Sample Analysis

To confirm the applicability of this proposed method, the f-MWCNTs were employed to determine the Pb^2+^ ions from real water samples. A standard addition method was used to verify the precision of the Pb^2+^ ion extraction from four different water samples such as drinking water, lake water, seawater, and tap water collected from Jeddah in Saudi Arabia. The percent extraction of the different amounts of Pb^2+^ ions (1.0, 5.0, and 10.0 mgL^−1^) using real water samples were determined (Table 3). The recovery test results showed that the extraction of Pb^2+^ ions from spiked water samples was acceptable and thus revealed that this method was suitable for analyzing real samples.

## 4. Conclusions

Herein, we functionalized MWCNTs by an acidic treatment. The as-obtained f-MWCNTs were characterized by XRD, BET, FESEM, XPS, XEDS, UV/vis, and FTIR spectroscopy. The detailed crystalline and morphological evaluations of XRD and FESEM demonstrated that the f-MWCNTs were almost tube-shaped, with carboxylic functional groups with typical average diameters of ~11.2 nm. The specific surface area was also investigated by a BET analysis and was found to be 222.45 m^2^/g by using the physical adsorption of nitrogen gases. The analytical efficiency of the f-MWCNT phase for the selective adsorption and detection of Pb^2+^ ions in an aqueous solution was studied. The Pb^2+^ ion static uptake capacity of 77.12 mgg^−1^ with f-MWCNTs adsorbent in aqueous media was reasonable. The sensitivity and linear dynamic ranges of the f-MWCNTs as a lead sensor were calculated from a calibration plot and were 0.8513 Lg^−1^ and 0–85.0 mgL^−1^, respectively. This proposed method provided reasonable results for the selective detection of toxic lead ions from spiked real water samples. Therefore, the method may be recognized as an efficient route in the selective detection of lead ions from complex matrices.

## Figures and Tables

**Table 1:** Selectivity study of the f-MWCNT phase adsorption toward different metal ions at pH 5.0 and 25.0 °C (*N* = 3).

Metal Ion	*q*_e_ (mgg^−1^)	*K_d_* (mLg^−1^)
Pb^2+^	4.99	7.13 × 10^5^
Cd^2+^	3.74	2.97 × 10^3^
Cr^3+^	3.69	2.81 × 10^3^
Cu^2+^	3.49	2.31 × 10^3^
Fe^3+^	3.27	1.89 × 10^3^
Zn^2+^	2.93	1.42 × 10^3^
Co^2+^	2.63	1.11 × 10^3^
Ni^2+^	0.91	2.22 × 10^2^

**Table 2:** Comparative study of lead metal ion uptake capacity with ICP-OES using various nanocomposite materials.

Materials	Methods	Detection Limit	Adsorption Capacity	References
MWCNTs–5-ASA	ICP-OES	0.25 ng mL^−1^	32.75 mg g^−1^	[76]
AC–EDA	ICP-OES	0.17 ng mL^−1^	-	[77]
EDA–MWCNTs	ICP-OES	0.35 ng mL^−1^	-	[78]
CuO–ZnO NCs	ICP-OES		82.66 mg g^−1^	[79]
SG-1,10-PhenanNTf_2_	ICP-OES	-	5.89 mg g^−1^	[80]
MWCNTs	ICP-OES	-	97.08 mg g^−1^	[81]
Co_3_O_4_–TiO_2_ NPs	ICP-OES	-	114.05 mg g^−1^	[82]

**Table 3:** Determination of Pb^2+^ at different concentrations in real water samples using 25.0 mg of f-MWCNTs (*N* = 3).

Samples	Added (mgL^−1^)	Not Adsorbed (mgL^−1^)	Extraction (%)
Tap water	1	0.02	98.10
	5	0.15	96.94
	10	0.45	95.49
Lake water	1	0.03	96.90
	5	0.20	95.92
	10	0.62	93.84
Seawater	1	0.04	96.40
	5	0.21	95.70
	10	0.82	91.85
Drinking Water	1	0.01	99.20
	5	0.10	97.96
	10	0.31	96.95

## Data Availability

Data will be available upon reasonable request.
